# Improving Risk Management in a Scaled Agile Environment

**DOI:** 10.1007/978-3-030-49392-9_9

**Published:** 2020-05-06

**Authors:** Eva-Maria Schön, Dirk Radtke, Christian Jordan

**Affiliations:** 6 University of Oslo, Oslo, Norway; 7 Monash University, Clayton, VIC Australia; 8 IT University of Copenhagen, Copenhagen, Denmark; 9 University of British Columbia, Vancouver, BC Canada; 10 University of Applied Sciences (HAW), Hamburg, Germany; 11 OTTO GmbH & Co KG, Hamburg, Germany

**Keywords:** Risk management, Agile methods, Agile software development, Scaling agile, E-commerce

## Abstract

Agile methods are designed for handling uncertainty as well as reducing risks in product development through transparency, inspection, and adaptation. Applying an effective risk management is in the nature of agile methods. However, when multiple agile teams work on the same product, a higher coordination effort is required and more formal practices are applied. The objective of this paper is to study how risk management can be improved in a scaled agile environment. Therefore, we conducted a case study in a large-sized ecommerce company and interviewed several project managers. The results show that there are differences for risk management in terms of two contexts. On the one hand, informal risk management is rated as good enough for one autonomous team. On the other hand, more formal approaches are needed, when several teams work on the same requirement. Furthermore, a tool for the support of risk management in a scaled agile environment is presented. We can conclude that hybrid development approaches consisting of agile practices and traditional practices, are beneficial, when several teams work in parallel.

## Introduction

Digital Transformation has an impact on the way an organization copes with challenges that arise, such as rapidly changing markets, evolving customer experiences, and disruptive technologies. In this context, many organizations have already recognized that agility is an important asset; they adopt agile methods like *Scrum* [1], *Kanban* [2], or *Extreme Programming* [3] for product development.

Agile methods have an impact on the organizational culture because of the agile values [4]. Those methods are designed to deal with complex, adaptive problems in the domain of emergence. People in agile environments need to probe first, then sense and then respond in order to handle the complexity [5]. Moreover, agile methods provide empirical approaches, which allow organizations to optimize predictability and control risk caused by iterative and incremental approaches [1]. In light of this, applying an effective risk management is in the nature of agile methods but is often implicitly handled.

In larger organizations, several teams often work on one product. For this reason, large-scale agile development becomes more important with the increasing spread of agile process models. When several agile teams work together on a product, there is a tension between the autonomy of a single team and the overall coordination of several teams. This often results in a considerable coordination effort caused by functional or technical dependencies among the teams [6]. Agile practices such as *Daily Standup Meeting, Kanban Board* or *Product Backlog* support the transparent handling of risks for an individual team. If several teams are working on the same requirement, these agile practices reach their limits. This can lead to a reduction in transparency and decisions cannot be taken on the basis of what is known. However, consistent practices and processes across teams and implementation of a common tool are seen as valuable in helping scaling agile methods [7]. Dingsøyr et al. [8] address the topic of large-scale agile development in a special issue. In particular, the special issue covers topics like the application of scaling frameworks, knowledge sharing, product ownership, and decision-making.

In this paper, we aim to address the research question (RQ): *How can the risk management in a scaled agile environment be improved?* Therefore, we conducted a case study in the ecommerce sector. The ecommerce sector is known for its rapid market development. Therefore, vendors need to react flexibly to changes and adapt early to new technologies to provide a unique customer experience.

The paper is structured as follows: Sect. 2 gives a brief overview of related work. Section 3 presents our research method and outlines the study context. Section 4 summarizes the key findings of our study, covering gaps and measures as well as presenting a new tool for risk management. Section 5 discusses the meaning of findings and limitations of this study. Finally, Sect. 6 concludes this work.

## Related Work

There is a difference between *uncertainty* and *risk*. Uncertainty can be perceived as both an opportunity and a threat [9]. On the other hand, a risk is characterized as an event that will have negative impacts when it occurs. In literature, some studies related to risk management in agile product development can be found. Analyzing the related work, we observe some similarities among the works. Authors investigate risk factors in agile environments and propose approaches to mitigate the identified risks.

Shrivastava and Rathod [10] study risk factors that affect the performance of distributed agile product development. In this context, they present a categorization of risks faced by practitioners as well as frequently used methods to reduce the impact of those risks. In 2017, Shrivastava and Rathod [11] propose a risk management framework, that consists of ranked risks for distributed agile development, its causes, and appropriate risk management approaches. Elbanna and Sarker [12] analyzed risk factors related to adopting agile development across multiple sectors like utilities, transportation, or financial services. In addition, they study their causes and consequences and present how different organizations deal with those risks. Tavares et al. [13] propose an extensive list of risk management practices for agile projects based on a literature review and relate them to subcomponents of agile methods, which are ranked by experts in accordance with their importance for risk management. Buganová and Šimícková [14] investigate the possibilities of the implementation of risk management in traditional and agile approaches to project management. They provide a comprehensive comparison between traditional, and agile and discuss the impact of risk management in the context of transportation companies.

## Research Method

The aim of this study is to investigate how risk management can be improved in a scaled agile environment. To this end, we used a case study in order to investigate this contemporary phenomenon in its context in industry [15].

### Study Context and Research Setting

The case study was carried out in 2018 in a large-sized ecommerce company, located in Germany. Today, the company generates more than 90% of its total sales through the online shop otto.de. In the past financial year, 7 million customers ordered online from OTTO. At peak periods, otto.de receives up to 10 new orders a second.

Currently, the team behind otto.de has 320 people working in 20 teams, of which 13 are functional teams that provide functions. Each team is purely vertical, consisting of different professions (Product Manager, Analyst, Usability, Interaction Designer, Quality, DevOps), and works in an interdisciplinary manner. They are fully responsible for the whole development process and operations in the cloud. The rest of the teams provide supporting services.

At the team level, there is complete freedom of choice for the team, as to which methods, practices, or variations are suited best. For some teams, the process is already ongoing for 200+ sprints. All core development teams are working on-site on a shared open plan office ground. The company heavily relies on face-to-face communication and tries out a flexible team assignment model, to support teams with laden backlogs.

Before conducting the study, we learned that there are two different contexts in which risk management must be considered. On the one hand, there is the context of *continuous product development,* in which agile teams implement features for the product in an autonomous manner. In this study, this context refers to the work of an autonomous team. This context is characterized by a decentralized, informal treatment of topics.

On the other hand, there is the context of the *cross*-*team project,* in which several agile teams work together to implement requirements. This context is characterized by explicit project constraints with regard to scope, timing, and budget. In comparison with the previous context, a more formal procedure for implementation is used.

### Data Collection and Analysis

**Fig. 1.**
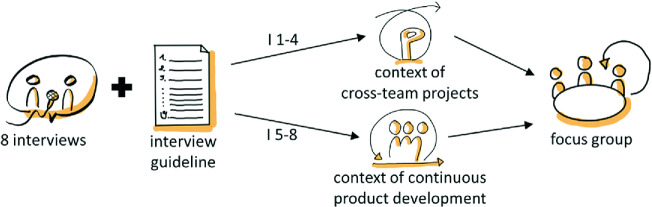
Research approach for analyzing the as-is situation

We started with an analysis of the as-is situation (see Fig. 1). Therefore, we gathered qualitative data by means of semi-structured interviews. The interview guidelines asked questions related to the roles and responsibilities of the participants, the as-is situation of risk management, types of risks, how risks are managed, and the measures taken. We closed the interview with an open question, so that participants had the option to express their further thoughts.

In sum, we conducted eight face-to-face interviews with a duration of 45 min each. We had four interviews with Production Leads, who are responsible for the context of *continuous product development*. Production Leads are only responsible for a single team. In addition, we had four interviews with *cross*-*team project* managers, who are responsible for the context of *cross*-*team projects*.

The interviews were documented in written form and aggregated into a result report. Then, the result report was discussed in a focus group in order to playback the results to the participants and to get feedback on the observations made so far. We discussed the identified problems concerning risk management. The participants then came up with measures that could be taken to remedy them.

## Results

**Fig. 2.**
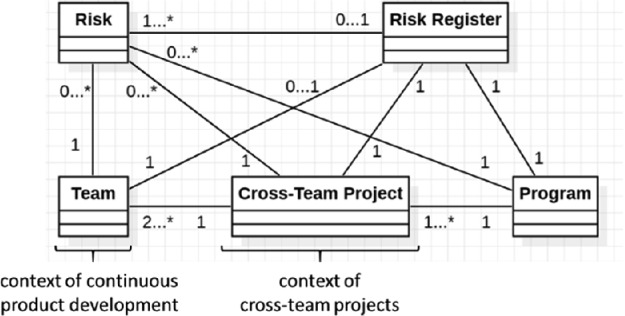
Overview of the improved risk management in a scaled agile environment

We will first provide an overview of the different scaling levels in order to present how we optimized the risk management in the scaled agile environment under study. For this purpose, we explain which measures have a positive effect on which level. Figure 2 shows the optimized context after the measures have been applied. In particular, it outlines how the handling of risks across the various scaling levels was optimized. The relationships are as follows: a program has one or many *cross*-*team projects*, whereas a *cross*-*team project* involves two or more teams. One team has none or many risks, which might be part of a risk register. In addition, one *cross*-*team project* can have none or many risks. A risk is documented by means of a template. In terms of the *cross*-*team project*, the handling of risks is improved by means of a mandatory risk register. The important risks of the *cross*-*team projects* are summarized into a risk register on the program level.

In the following, we will present our findings. We will outline how risk management is handled in the two different contexts. We will then describe the measures that are used to improve risk management.

### Continuous Product Development vs. Cross-Team Project

Table 1 outlines how risk management is conducted in accordance with the two different contexts (context of the *continuous product development* and context of *cross*-*team project*). Risk management in the context of *continuous product development* is an internal responsibility of the team. Since the individual teams work very autonomously and there is little dependency on other teams, the teams also deal with risks very differently. Nevertheless, there are some similarities. Implicit risk management is carried out through agile practices. In the daily meetings, urgent problems are discussed and, so, transparency about risks within the teams is created.

**Table 1.** Risk management in the context of *continuous product development* and in the context of *cross*-*team project*

**Context of the** ***continuous product development***	**Context of** ***cross-team project***
Informal risk management by agile approach	Formal risk management in the responsibility of dedicated project manager
Agile practices like daily standups, review meetings, and retrospectives promote a transparent management of risks	Explicit clarification of risk-related conditions and escalation instances during the project setup
Regular exchange among teams on best practices in risk management	Standardized approach for identification, management, and evaluation of risks, based on experience from previous, larger projects
	Regular exchange with relevant stakeholders on project risks

In comparison, more formal methods for risk management are used for the context of *cross*-*team projects*. However, the way in which this is done depends strongly on the project manager. In some cases, best practices from previous large-scale projects have been applied and enriched with the knowledge and experience of the particular project manager.

### Gaps and Measurements Related to Risk Management

After the results were discussed in the focus group (see Fig. 1), the participants came to the conclusion that informal risk management is sufficient for the context of continuous product development. In this context, therefore, only one workshop was conducted to exchange best practices for risk management in order to sensitize the teams to the topic and strengthen a conscious management of risks.

**Table 2.** Identified gaps in terms of risk management and measures to improve them

**Context**	***Continuous product development***	***Cross-team project***
Responsible for risks	Production lead, triade, team	*Cross*-*team project* manager, leading team
Identified gaps	For this context, informal risk management has been assessed as sufficient.	1. Clear, coordinated responsibility for risks is often lacking 2. Clear, coordinated responsibility for the implementation of measures is often lacking 3. Escalation instance for risks is unclear 4. Transparency with regard to risks from the perspective of the teams is often not given 5. Information flow at the project intersection *continuous product development* requires optimization
Measures	Best practices workshop on internal team risks	a) Optimization interface *cross*-*team project* - *continuous product development* (improves Gaps 1, 2, 4, 5) b) Optimization tooling for project management, and especially for risk management (improves Gaps 1, 2, 4, 5) c) Optimization project setup and management (improves Gaps 1, 2, 3)

However, the situation is different for the context of *cross*-*team project*. Some challenges could be identified (see Table 2), which can be summarized under the topics of responsibilities and transparency. During the focus group, appropriate measures were developed with which the identified challenges can be overcome. These are activities that are often associated with project management (see Table 2).

### Interface Cross-Team Project and Continuous Product Development

The optimization of the *interface between cross*-*team project and continuous product development* was carried out by strengthening the information flow in and out direction of the participating teams during the project duration. In detail, a regular communication of the status of individual risks was introduced, transparency in respect of upcoming project decisions (e.g. on risks), and furthermore, a close involvement of the experts from the participating teams in the technical discussions as well as risk assessment.

Moreover, biweekly program exchange meetings with the management were introduced. These meetings were about management support to exchange information on risks and not about reporting the status.

### Tooling for Risk Management

One of the measures to optimize the context *cross*-*team project* was the development of a suitable tool. The requirements for the tool were prioritized by the voting of the stakeholders (see 3.2). The following important requirements were identified: easy handling; filtering; transparency for all; exportability for a report; no mandatory fields; standardization using a template, whereby the principle *less is more* should be followed.

Then, three possible variants were tested. One based on *Excel* (Microsoft), one based on *Confluence* (Atlassian), and a search for suitable plugins on the market. After the evaluation by the stakeholders, the decision to go in for the Confluence-based solution was taken.

The tool is designed as follows: each project page in Confluence has its own subpage for risks. A button is used to capture new risks using a page template. The template includes the fields: topic, category, risk description, date of creation, date of update, probability of occurrence, impact, overall criticality score, measures, and person in charge. Thus, each risk is saved as an editable page. On a portfolio page there is a total cross-team projects risk register, which is automatically fed from the individual risks of the projects. This overall risk register can also be filtered, sorted and exported. Furthermore, the tool enables the aggregation of individual risks across several scaling levels (see Fig. 2) without the same risk being documented several times.

### Project Setup and Management

The *optimization project setup and management* included topics such as the explicit clarification of project responsibilities at the start of a *cross*-*team project*, clear communication of project responsibilities to stakeholders and ongoing support from program management.

For the project setup, an official kick-off meeting with the participating teams was introduced. In this kick-off meeting, information on the motivation of the project, the project goals, the control variables (e.g. timing, budget), the risk responsibilities, and decision-making groups were clarified.

In addition, project managers were regularly invited to the PMO. Participants in the PMO were the management circle and the organization team of the Level 2 Kanban board. In this meeting, the risk register of a cross-team project was discussed with the aim of identifying and clarifying the need for decisions. The clarification of the need for decisions may lead to a further discussion of a specific risk within the program exchange meeting with the senior management.

## Discussion and Limitations

Our results show that there is a need for action at *cross*-*team projects*, as we were able to identify some gaps here. The *cross*-*team project* requires more formal practices because several teams are involved, and it is a challenge to create transparency across team boundaries. The measures taken to improve risk management in the context of *cross*-*team project* (see Table 2) are often found as best practices in traditional project management. As a result, we can conclude that the pure application of agile methods and practices is not sufficient for a scaled agile environment. This is when hybrid process models come into play. Hybrid process models combine different methods and practices and are made up of natural process evolution, which is mainly driven by experience, learning, and pragmatism [16].

In summary, we achieved a proactive, conscious handling of risks because of the iterative actions in which the employees were actively involved. Moreover, we established a common understanding of risk management among the participants of the study. The main achievements for the company can be summarized as follows: support in operational risk management by means of a common tooling, standardized approach for risk management increases transparency, especially in the context of *cross*-*team projects* and assistance for the training of new employees.

Nevertheless, this study has some limitations. First, our findings are based on a single case study. So, the results might not be applicable to other cases on account of the specific context. Second, there might be a bias in the data collection procedure caused by missing audio or video recordings. However, we were able to mitigate this bias since we played back the summarized results of the interviews to the interview partners in a focus group. And third, the designed process for risk management could only be kept up by giving relevance to the autonomous teams because they decide whether to use it or not. This relevance could be given by management feedback.

## Conclusion

This paper presents findings from a case study conducted in a scaled agile environment in the ecommerce sector. The aim of our study was to improve the risk management within a company as well as improve the overall organizational design. In light of this, we examined two contexts, on the one hand, the context of *continuous product development* and, on the other, the context of *cross*-*team project*. We found that implicit risk management is sufficient for the former context, whereas a more formal risk management is required for the latter. To this end, we have worked out measures for the context of *cross*-*team project*, which address the identified challenges. In particular, the measures aim to improve the interface between *cross*-*team project*s and *continuous product development*, the tooling for project management, and especially for risk management across several scaling levels, as well as project setup and management.

Currently, we are working on an evaluation concerning the effectiveness of the applied measures. Therefore, we are planning several interviews with the participants of the study.
